# Curriculum development on the human rights of people with disabilities for future medical education: using a modified Delphi

**DOI:** 10.1186/s12909-021-02961-9

**Published:** 2021-10-29

**Authors:** Bomyee Lee, So-Youn Park

**Affiliations:** 1 Department of Medical Education and Medical Humanities, Kyung Hee University School of Medicine, Dongdaemun-gu, Seoul, 02447 South Korea; 2 Department of Policy & Research, Korea National Institute for Bioethics Policy, Jung-gu, Seoul, 04522 South Korea

**Keywords:** Students, Medical, Disabled persons, Right to health, Curriculum

## Abstract

**Background:**

In order for doctors to effectively provide medical services to patients with disabilities, an understanding of this population is necessary, along with the knowledge, attitudes, and technical abilities necessary to address health problems associated with each type of disability. One way of doing this is by educating doctors about disabilities and ensuring their frequent contact with people with disabilities while they are in medical school. Therefore, this study aimed to develop a systematic medical education curriculum to enhance doctors’ understanding of people with disabilities.

**Methods:**

The authors conducted a systematic literature review to develop and verify the basic framework of the educational content and curriculum. Two surveys were also developed using the Delphi method to evaluate the adequacy and necessity of educational topics. Items with a content validity ratio equal to or greater than the minimum value were considered valid. Survey panels comprised academic experts and health care practitioners who were working with people with disabilities. We conducted two surveys, one for a basic and the other for an advanced course, in which 13 to 16 respondents took part.

**Results:**

The authors selected 13 topics for the ‘Basic Introductory Course’ and included general educational content on the health rights of people with disabilities focused on improving students’ knowledge of disabilities. The authors also selected 12 topics for the ‘Care and Communication for Patients with Disabilities Course’ designed to improve students’ understanding of interviewing and communicating with patients with disabilities.

**Conclusions:**

In Korea, disability has received little attention in the medical curriculum to date. The curriculum developed in this study provides preliminary data for guiding future directions in medical education and developing specific support plans for an education that promotes people with disabilities’ health rights.

**Supplementary Information:**

The online version contains supplementary material available at 10.1186/s12909-021-02961-9.

## Background

In 2019, the number of registered disabled persons in South Korea was 2,618,000, accounting for about 5% of the total population [1] with 46.7% aged 65 years and older and 30.6% aged 50 to 64 years old [2]. The number of older adults with disabilities has increased due to an aging population; thus, there is an increased need to provide effective welfare and medical services for this group [3].

People with disabilities tend to have poorer health than people without disabilities due to the difficulties they face in receiving early treatment and preventive health management, as well as the increased prevalence of chronic diseases among people with disabilities and their difficulty paying for medical expenses [3–5]. Health care for people with disabilities in South Korea remains inadequate due to obstacles to physical access, such as financial burdens, a lack of convenient facilities, unsuitable medical equipment, and a lack of understanding of disability among health care workers [6]. Moreover, people with disabilities in South Korea experience significant inconvenience in accessing medical care due to health care workers’ poor understanding of disability characteristics, with 34.8% reporting they have experienced a lack of understanding and care, 26.8% mentioning a lack of amenities, and 14.1% claiming they have experienced difficulty communicating and accessing information [7].

Some global initiatives have attempted to address these problems, such as the European Commission’s (EC) European Disability Strategy 2010–2020, which established practical strategies to solve various problems at the national level. The EC acknowledged that people with disabilities have limited access to daily medical services due to inequality unrelated to their disabilities and attempted to ensure equal access to medical services and preventive health care across the board. The EC aimed to develop a policy to support the development of national educational programmes for health care workers and increase awareness of people with disabilities in medical schools [8].

Major medical schools in Western countries include various education curricula to help students gain an understanding of disabilities. For instance, Jacobs School of Medicine and Biomedical Sciences in the United States provides education on the knowledge, attitudes, and skills related to treating people with disabilities, which has been shown to have positive effects on students’ attitudes [9]. The Leeds School of Medicine in the United Kingdom provides seminars led by people with intellectual disabilities to help eliminate stereotypes and promote communication [10]. Also, McMaster University School of Medicine in Canada teaches communication with people with disabilities utilizing audio-visual content and a blended educational approach, including interaction with people with disabilities, which improves students’ confidence and proficiency in treating those with disabilities [11].

To improve the psychological accessibility of medical services for people with disabilities, South Korea implemented the ‘Act on Guarantee of Right to Health and Access to Medical Service for Persons with Disabilities’ in December 2017, stipulating that state and local governments should provide periodic education on the health rights of people with disabilities for health care workers, such as doctors, nurses, and medical technicians [12, 13]. In addition, the Ministry of Health and Welfare considered including education on understanding disability in university curricula for health care students [14]. However, despite the government’s plans, there is currently no formal curriculum related to understanding disability in Korea’s medical curriculum. Additionally, doctors felt insufficiently prepared and were not confident in prescribing treatments and assistive devices to children with disabilities [15] due to limited education, suggesting there is a need for education that deepens health care workers’ understanding of disability in medical schools [15, 16].

**Fig. 1:**
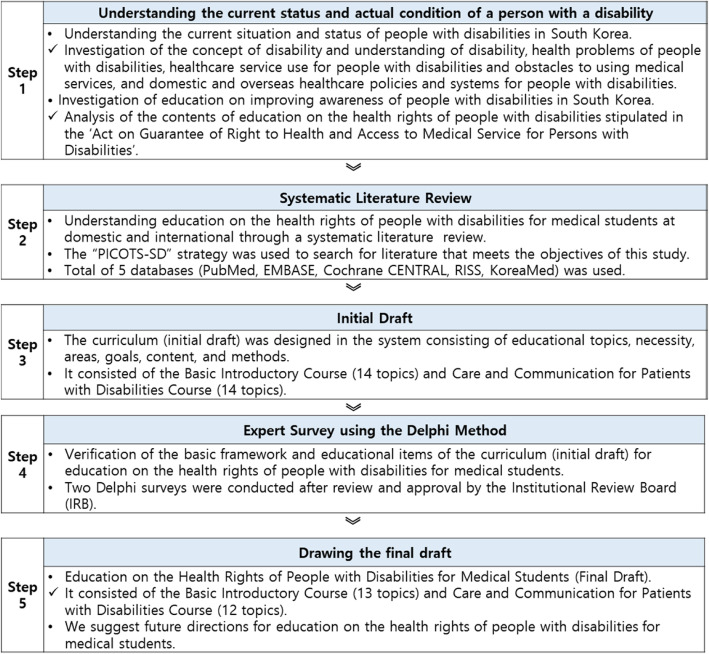
System for Conducting Research

It is required that doctors have an understanding of people with disabilities and the necessary knowledge, attitudes, and skills for treating various disabilities to provide effective medical services [17]. This can be acquired through education on disability and frequent contact with people with disabilities [18–20]. Therefore, this study aimed to develop a systematic curriculum to help medical students understand disabilities and better interact with patients with disabilities. A framework of the education curriculum on the health rights of people with disabilities for medical students was prepared after conducting a systematic review of this type of education programme both domestically and abroad. The validity of the proposed education curriculum was verified using a modified Delphi method. This study was conducted according to a total of five steps (Fig. 1) towards developing a curriculum for the right to health of people with disabilities.

## Methods

### Understanding the current status and actual condition of a person with disabilities

We investigated the concept and public understanding of disability, the current status of health problems among people with disabilities, the current health services available for them, the factors of medical use, and the domestic and international health care policies and systems in place for people with disabilities.

### Systematic literature review

By systematically considering the current status of education on the health rights of people with disabilities in both domestic and international medical schools, this study conducted a systematic literature review to identify recent research trends and analyse effective educational content. We systematically reviewed current medical education resources for people with disabilities both domestically and abroad to develop a basic curriculum framework and identify educational items using the process developed by Kim and colleagues [21]. The ‘PICOTS-SD’ strategy was used to search for literature that met the study objectives. A total of five databases (i.e., PubMed via MEDLINE, EMBASE via Elsevier, Cochrane CENTRAL, RISS, and KoreaMed) were used. Papers published before April 17, 2020, were considered.

**Fig. 2:**
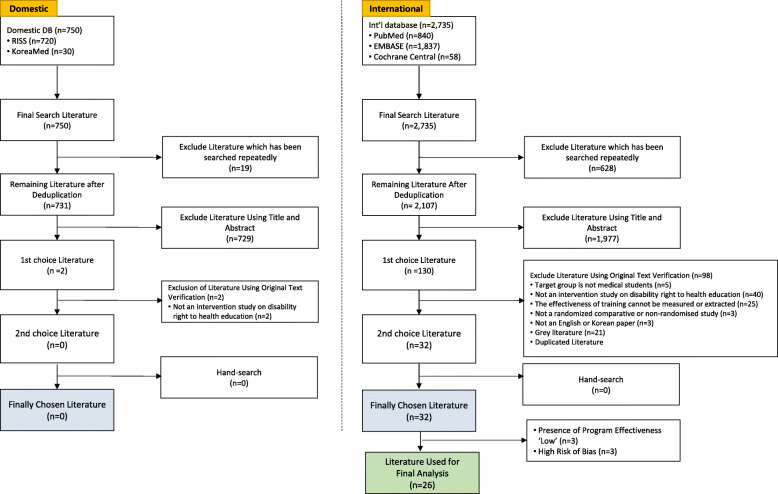
PRISMA diagram

In order to analyse the educational contents of the right to health for people with disabilities for medical students, three literature sources in which all measurement variables were not statistically significant (whether or not the programme effect was ‘low’) were excluded from the analysis, among 32 literature sources finally selected through the systematic literature review process. In addition, three literature sources with a high risk of bias in ‘incomplete outcome data’ and ‘selective reporting’ were also excluded from the analysis. Among the 26 analysed literature sources, none of them was written in Korean. The PRISMA diagram of systematic literature review is as shown in Fig. 2.

### Initial draft

Educational items were divided into the ‘Basic Introductory Course’ and ‘Care and Communication for Patients with Disabilities Course’. The initial draft contained 14 educational topics each, including necessity, area, goal, content, and method. The ‘Basic Introductory Course’ contained general educational content on the health rights of people with disabilities and focused on improving students’ knowledge. The ‘Care and Communication for Patients with Disabilities Course’ was designed to improve health care professional’s interview and communication skills when treating patients with disabilities based on the content learned in the ‘Basic Introductory Course’.

### Expert survey using the Delphi method

The selection of an expert panel is very important because the results of the Delphi survey depend critically on expert knowledge, opinions, and intuition [22].

Since the Delphi survey was largely divided into the ‘Basic Introductory Course’ and ‘Care and Communication for Patients with Disabilities Course’, it was necessary to select a different expert panel for each part. The Basic Introductory Course contains overall educational contents on the right to health for people with disabilities and is mainly designed to improve students’ level of knowledge. Therefore, not only academic experts in rehabilitation medicine, preventive medicine, social welfare, among others, but also practitioners of associations and organisations related to people with disabilities were included, as it was necessary to select a panel of experts from as diverse fields as possible. Furthermore, three experts with physical disabilities were included in the panel to obtain more detailed advice on disability-related issues. The Care and Communication for Patients with Disabilities Course was composed with the purpose of improving interview and communication skills with disabled patients based on what was learned through the basic introductory course. Therefore, the panel was mainly composed of the following in order to produce good doctors: professors of the Department of Rehabilitation Medicine who treat patients with disabilities, and professors in the field of medical education who are in charge of developing medical education courses, clinical skills education, and humanities and social medicine education. The expert panel was selected through purposive sampling from assistant professors in related departments and managers of associations and organisations related to people with disabilities at executive positions.

Two surveys with different panels for each course were conducted via e-mail between August and September 2020. For the ‘Basic Introductory Course’, 16 and 15 people responded to the first and second surveys, respectively, while for the ‘Care and Communication for Patients with Disabilities Course,’ 14 and 13 people responded, respectively. For each cluster, the experts in the first and second surveys were the same person, and one person in each cluster did not respond to the second survey.

The first Delphi survey evaluated the ‘necessity’ and ‘adequacy’ of each potential educational item on a 4-point Likert scale (1 = *not necessary*/*not adequate*, 4 = *very necessary/very adequate*). ‘Necessity’ indicated whether the item was essential to medical students, while ‘adequacy’ indicated whether the educational content and method were adequate for the educational topic. For the second Delphi survey, we revised the questionnaire based on the results of the first survey and sent them to the expert panel for re-evaluation of items upon which agreement was lacking in the first survey. Based on the results of the second survey, we composed the final draft of the curriculum. We analysed the survey results by calculating the content validity ratio (CVR) for each item. The CVR suggested by Lawshe [23] is calculated by considering the number of cases and the number of respondents who provided valid responses. In other words, the minimum value varies depending on the number of respondents. For example, if the number of respondents is 13 and the CVR value is .54 or higher, content validity is recognised (Table 1). In principle, indicators were selected only if the CVR value was minimum or greater in both ‘necessity’ and ‘adequacy’ of the indicator.

**Table 1:** Minimum CVR according to the number of responded panels

Responded number of panels (N)	Minimum CVR	Applied to this study
10	.62	
11	.59	
12	.56	
13	.54	‘Care and Communication for Patients with Disabilities Course’, as of the second survey
14	.51	‘Care and Communication for Patients with Disabilities Course’, as of the first survey
15	.49	‘Basic Introductory Course’, as of the first survey ‘Basic Introductory Course’, as of the second survey
20	.42	

^a^e [23]

### Drawing the final draft

Based on the results of the second Delphi survey, the curriculum (final draft) for the right to health for people with disabilities was formed. This curriculum proposes possible future directions for medical education to ensure the health rights of people with disabilities.

## Results

### Initial draft

Our review of previous studies revealed that various educational methods (e.g., blended learning incorporating theoretical and practical education) and educational strategies (e.g., blended education including all school-based, community-based, and clinical-based practice training) have been trialled [24–26]. Thus, we organised the draft curriculum using a blended education method, including school-based lectures as well as clinical practices and community-based activities.

In addition, we found that when people with disabilities and their families or guardians participated in educational programmes and interacted directly with students, this positively influenced students’ attitudes towards people with disabilities [27, 28]. Therefore, our initial draft included the participation of people with disabilities and their families and guardians in the education curriculum. Furthermore, we found that previous education methods aimed at improving knowledge about disabilities and improving the interview and communication skills of those interacting with people with disabilities [29–31]. Thus, we followed a similar approach in our curriculum.

Moreover, international studies suggested the necessity of some common traits in future education. As previous studies have focused on one-time or short-term education, long-term effects on students’ knowledge, attitudes, and skills were not determined in the proposed curriculum. Medical students who participated in such short-term education programmes stated that they were insufficient, and that additional education was required [24, 25, 31–36]. Therefore, long-term education programmes on the health rights of people with disabilities are necessary. We developed 14 educational topics in the initial drafts of the ‘Basic Introductory Course’ and the ‘Care and Communication for Patients with Disabilities Course’ to address this need. Finally, as the characteristics of people with disabilities vary according to disability type, we considered comprehensive medical education on various disabilities to be necessary [24, 25, 30]. As such, our curriculum included various disabilities: physical disabilities, hearing impairment, visual impairment, and developmental disorders.

### Analysis of expert survey results using Delphi method

#### Expert panel characteristics

The gender distribution of the expert panel was relatively uniform, and more than half of the respondents stated that they had between 10 and 20 years of experience in their field. The panel for the ‘Basic Introductory Course’ included more practitioners from associations and organisations for people with disabilities, while the ‘Care and Communication for Patients with Disabilities Course’ panel included more academic experts in rehabilitation medicine (Table 2).

**Table 2:** General Characteristics of the Delphi Expert Panel

Division		First survey	Second survey
		*n* (%)	*n* (%)
**- Basic Introductory Course**			
Sex	Men	9 (56%)	8 (53%)
	Women	7 (44%)	7 (47%)
Specialty and major	Academia	7 (44%)	6 (40%)
	- Rehabilitation medicine	4 (57%)	4 (67%)
	- Social welfare	2 (29%)	2 (33%)
	- Preventive medicine	1 (14%)	0 (0%)
	Field (associations and organisations)	9 (56%)	9 (60%)
Career	Fewer than 10 years	3 (19%)	3 (20%)
	10 to 20 years	11 (69%)	10 (67%)
	More than 20 years	2 (13%)	2 (13%)
Total		16	15
**- Care and Communication for Patients with Disabilities Course**			
Sex	Men	8 (57%)	7 (54%)
	Women	6 (43%)	6 (46%)
Specialty and major	Academia	12 (86%)	11 (85%)
	- Rehabilitation medicine	9 (75%)	9 (82%)
	- Medical education	2 (17%)	2 (18%)
	- Preventive medicine	1 (8%)	0 (0%)
	Field (associations and organisations)	2 (14%)	2 (15%)
Career	Fewer than 10 years	1 (7%)	1 (8%)
	10 to 20 years	11 (79%)	10 (77%)
	More than 20 years	2 (14%)	2 (15%)
Total		14	13

^a^ Percentages (%) are rounded to the nearest whole number

#### First survey results

The survey for the ‘Basic Introductory Course’ contained eight items with a CVR value of 0.49 or higher for necessity and adequacy (Table 3). These items were not re-evaluated in the second survey. However, five items required re-evaluation (Table 3). One item (disability experience education II) was deleted as it had a low CVR value for both necessity and adequacy (deleted educational contents are organised in separate files, Supplementary File 2). Experts in the first survey noted that this item ‘can make students feel more negative about people with disabilities and that education should teach students that inconveniences are caused by social and environmental problems’.

**Table 3:** Results of the First and Second Delphi Surveys

	Topic	First Delphi*			Second Delphi**		
		CVR		Results	CVR		Results
		Necessity	Adequacy		Necessity	Adequacy	
- Basic Introductory Course							
1	Concept of disability and understanding of disability	1.00	1.00	Selected	–	–	Selected (first)
2	Definition and characteristics of disability	0.75	0.88	Selected	–	–	Selected (first)
3	Laws and policies related to people with disabilities at home and abroad	0.75	0.63	Selected	–	–	Selected (first)
4	Health of people with disabilities	1.00	1.00	Selected	–	–	Selected (first)
5	Obstacles to using medical services I	1.00	0.88	Selected	–	–	Selected (first)
6	Obstacles to using medical services II (special lecture by people with disabilities)	1.00	0.88	Selected	–	–	Selected (first)
7	Understanding assistive technology devices for people with disabilities	0.63	0.38	Reevaluation required	0.63	0.73	Selected (second)
8	Disability experience education I	0.25	0.25	Reevaluation required	0.73	0.60	Selected (second)
9	Etiquette for various disabilities	0.63	0.50	Selected	–	–	Selected (first)
10	Communication with people with disabilities	0.88	0.63	Selected	–	–	Selected (first)
11	Disability experience education II	0.00	0.00	Deleted	–	–	Deleted (first)
12	Community service	0.63	0.38	Reevaluation required	0.63	1.00	Selected (second)
13	Meeting people with disabilities in the community	0.25	0.38	Reevaluation required	0.87	0.73	Selected (second)
14	Research related to people with disabilities	0.50	0.25	Reevaluation required	0.50	0.73	Selected (second)
- Care and Communication for Patients with Disabilities Course							
1	What is communication?	0.71	0.71	Selected	–	–	Selected (first)
2	Building rapport with patients with disabilities	1.00	0.86	Selected	–	–	Selected (first)
3	Communication with patients with disabilities: type I (patients with visual impairment)	1.00	0.86	Selected	–	–	Selected (first)
4	Communication with patients with disabilities: type II (patients with hearing impairment)	0.86	0.71	Selected	–	–	Selected (first)
5	Communication with patients with disabilities: type III (patients with developmental disorders)	0.86	0.71	Reevaluation required	1.00	0.85	Selected (second)
6	Patient consent I (theory)	0.71	0.57	Reevaluation required	1.00	0.85	Selected (second)
7	Patient consent II (consent for CT scan)	0.29	0.29	Deleted	–	–	Deleted (first)
8	Patient consent III (organ donation)	−0.14	−0.29	Deleted	–	–	Deleted (first)
9	Basic principles of treatment of patients with disabilities	1.00	1.00	Selected	–	–	Selected (first)
10	Treatment of patients with disabilities: type I	1.00	1.00	Selected	–	–	Selected (first)
11	Treatment of patients with disabilities: type II	0.57	0.57	Selected	–	–	Selected (first)
12	Meeting patients with disabilities	0.43	0.43	Reevaluation required	0.54	0.69	Selected (second)
13	Mock interviews using standardized patients I (patients with visual impairment)	0.71	0.57	Selected	–	–	Selected (first)
14	Mock interviews using standardized patients II (patients with intellectual disabilities)	0.86	0.57	Selected	–	–	Selected (first)

* minimum CVR: 0.49 (first and second survey)

** minimum CVR: 0.51 (first survey), 0.54 (second survey)

The survey for the ‘Care and Communication for Patients with Disabilities Course’ had nine items with a CVR value of 0.51 or higher for both necessity and adequacy (Table 3). These items were not re-evaluated in the second survey. We re-evaluated two items with a CVR value of 0.51 or higher for necessity and adequacy (consensus reached) that were modified by referring to common expert opinions and one item with a CVR value lower than 0.51 for necessity and adequacy (Table 3). Two items (patient consent II and patient consent III) were deleted as they had low CVR values for necessity and adequacy (deleted educational contents are organised in separate files, Supplementary File 2). Experts in the first survey stated that it would be difficult to plan and conduct meetings with patients with disabilities in busy hospitals and suggested ‘replacing clinical-based education with community-based education’. Experts also noted that role-playing activities may be inappropriate for developmental disorders, as they require an in-depth understanding of the disability.

#### Second survey results

The five questions that needed re-evaluation in the ‘Basic Introductory Course’ were revised by reflecting the opinions of the expert panel in the first survey as much as possible. For example, for the topic ‘understanding assistive technology device for people with disabilities’, the educational content was modified so that the policy approach (e.g., the assistive device purchase-related support system and assistive device application procedure), along with the assistive technology approach, could be dealt with in combination. The educational theme of ‘disability experience education I’ reflects the opinion that disability-related education may cause students to have more negative thoughts towards people with disabilities. Consequently, the educational content was revised and supplemented to emphasize the idea that, more than a source of discomfort, disability is a social issue. The students were also encouraged to discuss the issued faced by people with disabilities. The topic ‘community service’ education includes not only introducing the concept of community service, but also educating students about how actual community resources are related to each other and showing good examples of cooperation with the local community. In addition, the content of volunteering at disability-related organisations, such as the general welfare centre for people with disabilities, was added to the education topic ‘meeting people with disabilities in the community’. Lastly, under the topic ‘research related to people with disabilities’, educational contents were added to introduce various data sources for research on disabilities to students prior to conducting the research.

The three items that needed re-evaluation in the ‘Care and Communication for Patients with Disabilities Course’ were revised by reflecting the opinions of the expert panel in the first survey as much as possible. For example, for ‘communication with patients with disabilities: type III (patients with developmental disorders)’, the education topic reflects the opinion that role-play classes are not appropriate as an educational method because it is necessary to understand the disability at a considerable level in order to imitate the characteristics of the developmentally disorders. Therefore, the educational method was modified by conducting discussions through case studies and sharing cases by special lecturers who frequently encounter people with developmental disorders. In the topic ‘patient consent I (theory)’, the case of obtaining a consent form for CT scan for patients with intellectual disabilities was added to the educational content. In addition, the educational theme ‘meeting patients with disabilities’ replaced clinical-based education, and it was reconfigured to carry out medical service activities in connection with disability-related groups and institutions in the local community.

According to the second Delphi survey, all five re-evaluated items for the ‘Basic Introductory Course’ had a CVR value of 0.49 or higher, indicating high validity, and were included in the final draft. Likewise, all three re-evaluated items for the ‘Care and Communication for Patients with Disabilities Course’ had a CVR value of 0.54 or higher, indicating high validity, and were included in the final draft (Table 3).

### Final draft

We revised the curriculum contents based on the survey results. We selected 13 topics for the ‘Basic Introductory Course’ and 12 topics for the ‘Care and Communication for Patients with Disabilities Course’ (Supplementary Information, Additional file 1 [37, 38]).

## Discussion

Previous studies have found panel discussions that included patients with disabilities to be an effective and meaningful educational method for medical students [24, 39]. Contact-based education through direct encounters or interactions helps medical students build communication skills and gain confidence in treating people with disabilities. Clinical practice and community-based education in rehabilitation hospitals could provide students with an opportunity to engage in real contact with people with disabilities [28]. In addition, education programmes that use standardised patients help medical students face real-world situations, preparing them for the reality of a clinical environment in a space where students can learn from their mistakes, receive feedback, and reflect [40]. However, there are some practical obstacles to using standardised patients. First, it may be difficult to accurately portray subtle differences in characteristics exhibited by people with disabilities. In addition, stereotypes may be inadvertently reflected, which can negatively affect students and reduce the effectiveness of education. Therefore, real patients with disabilities should be included in education programmes whenever possible [41].

Additionally, an appropriate form of evaluation is required to determine whether medical students’ knowledge, attitude, and skills related to the treatment of people with disabilities have improved. Education should focus on improving students’ skills, such as treatment and communication skills, when caring for patients with disabilities, rather than simply providing knowledge. Such skills can be evaluated using clinical performance tests, observation and feedback through video recording, self-reporting, and student discussion [30, 34]. If specific goals or evaluation criteria are not presented to students, they may lose interest and avoid active participation [29, 42]. Therefore, appropriate evaluation methods should be considered when designing educational programmes [43].

Furthermore, improper education on the health rights of people with disabilities can instil negative perceptions of disability. Theoretical education may emphasise the medical aspects of disability, such as disability characteristics, onset, and causes. Therefore, education must also be based on social and environmental rather than solely medical perspectives, focusing on preventive health care rather than on the health problems of people with disabilities to prevent the development of prejudice.

Moreover, education must be continuous and educational methods that enhance student motivation should also be considered. As noted above, the long-term effects of short-term education on student attitudes and behaviour are unknown [11, 24, 31, 42]. Furthermore, as positive attitudes towards people with disabilities may deteriorate over time [29, 44, 45], continuous education may be required for practicing doctors. Education with low levels of response and participation may be less effective and may feel like a burden, perceived as merely a credit requirement by students. Some medical schools offer education on the health rights of people with disabilities as an elective course. The effectiveness of such electives is uncertain, as students who choose these courses are likely to already have an interest in or a positive attitude towards people with disabilities [26, 28, 31]. However, the elective nature of these courses may simultaneously increase student interest and participation.

Finally, medical education on disabilities and skills for caring for people with disabilities must have its basis in a systematic curriculum that ensures that graduates have the required attitudes and skills [25, 34]. However, including such courses as regular curricula in medical schools may be challenging. Several practical problems may arise, such as obtaining the approval and budget for a new educational programme, organising the appropriate faculty, and establishing the necessary community network [26]. These issues require universities and communities to make a collective effort. Awareness of the necessity of education for the health rights of people with disabilities must increase among university faculty members, government policy makers, and the community as a whole.

### Limitations

This study divided the curriculum of medical education on the health rights of people with disabilities into the ‘Basic Introductory Course’ and the ‘Care and Communication for Patients with Disabilities Course’. This paper presents details of the educational topics, needs, areas, goals, content, and methods of these courses. However, the effectiveness of the curriculum proposed by this study has not yet been evaluated. Future research should test the effectiveness of this curriculum by applying it in a real educational context.

## Conclusion

This study offered a roadmap to a medical education curriculum to teach medical students how to treat and work with patients with disabilities. The proposed curriculum would allow medical students to understand the causes of the difficulties faced by people with disabilities in accessing medical services and to consider possible solutions. This curriculum is also likely to help medical students acquire professional skills and attitudes, as well as increase their sense of social accountability when treating patients with disabilities.

Furthermore, this study emphasized the need for education on the health rights of people with disabilities among medical students in South Korea, where there is currently a lack of awareness. To improve access to medical services for people with disabilities, South Korea aims to include modules on the understanding of disability in its medical schools curricula [14]. The curriculum proposed in this study is in line with this national policy. It offers a foundation for the development of mid- to long-term education in this field. In addition, it would facilitate cooperation with people with disabilities living in the community, a method that has not been considered in medical school curricula to date. This would enable people with disabilities to be perceived as fellow members of society. The learning opportunities provided by this curriculum will develop medical students’ senses of social accountability and help them to actively engage in and establish working partnerships with patients with disabilities.

## Supplementary Information

**Additional file 1: Supplementary File 1.** Education on the Health Rights of People with Disabilities for Medical Students (Final Draft). **Supplementary File 2.** Education contents that were not selected by experts.

## Data Availability

NA

## Abbreviations

CVR: Content validity ratio
EC: European Commission
