# Supplementary material for: Curriculum development on the human rights of people with disabilities for future medical education: using a modified Delphi
Source: BMC Med Educ. 2021 Oct 29;21:548. doi: 10.1186/s12909-021-02961-9 (PMC8555282; doi:10.1186/s12909-021-02961-9)
Supplement: Supplementary file 1 — Additional file 1: Supplementary File 1. Education on the Health Rights of People with Disabilities for Medical Students (Final Draft). Supplementary File 2. Education contents that were not selected by experts. [file 12909_2021_2961_MOESM1_ESM.docx]

**Supplementary File 1.** Education on the Health Rights of People with Disabilities for Medical Students (Final Draft)

- Basic Introductory Course (Final Draft)

|  | Topic | Necessity | Area | Goal | Content | Method | Reference |
| --- | --- | --- | --- | --- | --- | --- | --- |
| 1 | Concept and understanding of disability | - A critical perspective is required for the medical model-centered view of disability. | Knowledge | - Students can explain how the rights of people with disabilities have been socially redefined by looking at the main trends of the disability rights movement at home and abroad. | - Understanding of disability   - Concept of disability and flow of change in perception of disability  - Medical cause ⇒ social and environmental factors  - International Classification of Functioning, Disability, and Health  - UN Convention on the Rights of Persons with Disabilities   - Main historical flows and features of the disability rights movement   - History of independent living movement   - Representative prejudices and stereotypes against people with disabilities - Proper expression of the disability | - School-based   - Lectures on the concept of disability and historical flow  - Discussion of questions such as defining disability and what should be included as a disability | [11, 24, 25, 26, 28, 34] |
|  |  |  | Attitude | - Students have a critical attitude towards disability issues through understanding the different perspectives of the medical and social models of disability. |  |  |  |
| 2 | Definition and characteristics of disability | - Efforts are required to understand people with disabilities, taking into account the disabilities’ characteristics based on a proper understanding of the definition of disability. | Knowledge | - Students can explain the definition of disability, the type of disability, and the criteria for determining the disability stipulated in the ‘Act on Welfare of Persons with Disabilities’ in South Korea. - Students can explain the status of registered people with disabilities in South Korea and the characteristics, cause, and onset time of various disabilities. | - Definition of disability - Domestic disability classification and criteria   - Type and severity of disability  - Registration and screening system for people with disabilities   - Status of registered people with disabilities in South Korea (statistics) - Characteristics, cause, and time of onset of various disabilities  (※Lectures avoiding emphasis on medical aspects of disability characteristics) - Sex characteristics, such as maternity rights - Quality of life of people with disabilities, such as education, housing, income, and occupation | - School-based   - Lecture on the definition and characteristics of disability | [3, 11, 12, 13, 14] |
| 3 | Laws and policies related to people with disabilities at home and abroad | - It is necessary to recognize that people with disabilities are also citizens who have the right to live as human beings and that this right should be guaranteed to them. - To guarantee the basic rights of people with disabilities, a comprehensive understanding of the flow of the policies being promoted at home and abroad is required. | Knowledge | - Students can explain the purpose, general contents, and main provisions of major laws and policies related to people with disabilities - Students can understand the lawful rights that people with disabilities should have and discuss the provisions that need to be improved. | ※In education, rather than a simple listing of laws, the background of legislation should be introduced.   - Overseas   - World Health Organization (WHO) Global Disability Action Plan  - European Union (EU) Disability Strategy  - United Nations Convention on the Rights of Persons with Disabilities   - Domestic   - Background, ideology, purpose, main contents, and business of the enactment of the ‘Act on Guarantee of Right to Health and Access to Medical Service for Persons with Disabilities’  - Comprehensive Policy Plan for People with Disabilities  - ‘Act on Welfare of Persons with Disabilities,’ ‘Act on the Prohibition of Discrimination against Persons with Disabilities, Remedy against Infringement of Their Rights, etc.’. | - School-based   - Lectures on laws and policies related to people with disabilities at home and abroad  - Presenting and discussing cases with limitations and contradictions among the systems and services that can be used within laws related to people with disabilities | [12, 24, 25, 28, 34, 40] |
| 4 | Health of people with disabilities | - It is necessary to understand the health status of people with disabilities and the necessity of healthcare to promote health. - Education on the importance of preventive health care is necessary to prevent prejudiced beliefs that it is difficult for people with disabilities to enjoy a healthy life. | Knowledge | - Students can explain the current status of health problems for people with disabilities in South Korea, use of healthcare services for people with disabilities, and preventive healthcare for people with disabilities. - Students can explain the status and importance of preventive healthcare. | - Health of people with disabilities in South Korea   - Chronic disease prevalence, obesity rate, subjective health status, and depression rate  - Lack of preventive healthcare and occurrence of secondary disorders, such as bedsores, urination and defecation disorders, sleep disorders   - Use of healthcare services for people with disabilities   - National health examination screening rate, cancer examination screening rate, hospitalization rate, medical expenses   - The status and importance of preventive healthcare for people with disabilities   - Health checkup, smoking cessation, abstaining from alcohol, regular eating habits, and health behaviors such as aerobic physical activity | - School-based   - Lectures on the health of people with disabilities in South Korea | [3, 4, 5, 6, 9, 11] |
| 5 | Obstacles to using medical services I | - A comprehensive understanding of the barriers experienced by people with disabilities in using medical services is required. - To improve accessibility to medical care for people with disabilities, medical students who will become healthcare workers in the future must be educated about desirable doctors’ attitudes. | Knowledge | - Students can explain the obstacles to the use of medical services for people with disabilities, including environmental, structural, and procedural barriers. | - Obstacles to using medical services   - Environmental barriers: transportation restrictions, medical institution facilities, and inspection equipment designed for people without disabilities that are inconvenient for people with disabilities  - Structural barriers: the economic level of patients with disabilities, level of health insurance benefits  - Procedural barriers: reservation system, long waiting time, lack of healthcare workers’ knowledge and awareness of disabilities, prejudice about patients with disabilities | - School-based   - Lectures on the obstacles to using medical services for people with disabilities  - Discussion of solutions to improve medical accessibility through specific problematic cases | [34, 40] |
|  |  |  | Attitude | - Students have a critical view of prejudices related to patients with disabilities. |  |  |  |
| 6 | Obstacles to using medical services Ⅱ  (special lecture by people with disabilities) | - Students should have an empathetic attitude towards people with disabilities, obtained through listening to actual stories of people with disabilities about various difficulties that they experience while using medical services. | Attitude | - Students have an empathetic attitude towards situations that people with disabilities experience when using medical services. | - Special lecturers should address the following:   - Sharing actual experiences of using medical services (positive and negative aspects, how they felt at that time)  - Introducing best practices for medical staff to interact effectively with people with disabilities  - Desirable attitudes of healthcare workers | - Community-based   - Meeting actual patients with disabilities and lectures by people with disabilities  - Medical staff treating people with disabilities provide special lectures on the treatment experience and precautions for treating people with disabilities | [11, 24] |
| 7 | Understanding assistive technology devices for people with disabilities | - It is necessary to have a general understanding of assistive technology devices for people with disabilities in daily life as well as educational and vocational activities, which enable them to live independently. | Knowledge | - Students can explain the types of assistive technology devices mainly used by people with disabilities. - Students can discuss what assistive technology devices are required based on the characteristics and needs of various types of disabilities and living environments. - Students can explain the support system related to the purchase of assistive equipment and the procedure for applying for assistive equipment. | - Introduction to various types of assistive technology devices   - Mobile assistive devices  - Communication aids  - Audio and visual aids  - Assistive devices for everyday life   - Understanding universal design and introduction of universal design medical equipment - Cases demonstrating the improvement of everyday life by using assistive devices - Support system related to the purchase of assistive equipment and the procedure for applying for assistive equipment | - School-based   - Lectures on the concepts and types of assistive technology devices  - Group discussion and presentations on assistive technology devices required for various disabilities | [34] |
| 8 | Disability experience education | - Wheelchair experience education and visual impairment experience education help with understanding disability and the importance of providing amenities in consideration of people with disabilities. - Students should be able to think of ways to improve facilities and environments inconvenient for people with disabilities. | Knowledge | - Students can explain the structure and use of wheelchairs and canes. - Students understand the importance of amenities for people with disabilities. | - Students understand facilities and environments that people with disabilities utilize - The structure and terminology of wheelchairs, how to move a wheelchair, how to move around obstacles, how to assist people with disabilities in wheelchairs - The structure and usage of canes, individual and guided walking methods for those with visual impairment, obstacle identification, and movement method   ※ To avoid the prejudice that disability is uncomfortable and pitiful, after experiential education, discussion of methods to improve facilities and environments inconvenient for people with disabilities should be conducted. It is important to guide students to approach disability experience education seriously. | - Community-based   - Visiting community centers with wheelchair and road traffic experience facilities, dark rooms, and virtual reality (VR) facilities for experience education   - School-based   - Discussion of methods to improve facilities and environments inconvenient for people with disabilities. | [26, 41] |
|  |  |  | Attitude | - Students have an empathetic attitude towards people with disabilities regarding the inconvenience of using facilities and environments. |  |  |  |
| 9 | Etiquette for various disabilities | - Students should have the attitude and values of living with people with disabilities, obtained through understanding the diversity of disabilities and learning etiquette for dealing with people with disabilities. | Knowledge | - Students can explain the main contents of etiquette for people with disabilities by disability type. | - The following etiquette should be included in education:   - Physical disability: Assisting with movement in places with stairs and escalators, eating with people with disabilities, using a door or elevator, talking to people with disabilities in a wheelchair, pushing a wheelchair  - Facial disfigurement: avoiding triggering questions or words that remind people with disabilities of the time of the accident  - Visual impairment: guiding, talking with the blind person, reading texts  - Hearing impairment: gestures and facial expressions when speaking with hearing-impaired people, communication with hearing impairment  - Developmental disorders: using honorific language according to chronological age | - School-based   - Lectures on etiquette by disability type | [11, 24, 26, 31] |
|  |  |  | Attitude | - Students can minimize the sense of distance to people with disabilities based on the understanding etiquette by disability type as well as respond correctly to requests for help. |  |  |  |
| 10 | Communication with people with disabilities | - Students should have the attitude and values of living with people with disabilities, obtained through learning communication methods for people with hearing impairment, speech impairment, and developmental disabilities who have difficulty communicating. | Knowledge | - Students can explain how to communicate with people with disabilities and note precautions. | - Communication with people with disabilities   - Main characteristics of the communication disorder category  - Communication difficulties and precautions  - Communication method and skills for various types of disabilities  - Complementary and alternative communication: necessity, meaning, method, type | - School-based   - Lectures on communication methods and precautions for people with disabilities | [11, 26, 33] |
|  |  |  | Attitude | - Students are confident in smoothly talking to people with disabilities. |  |  |  |
| 11 | Community service | - A comprehensive understanding of the types of community services for people with disabilities is required. | Knowledge | - Students can describe the types of community services and their delivery system for people with disabilities. | - Types of community services for people with disabilities and how to use them   - Economic and income support  - Medical, rehabilitation, and health support  - Psychological, social, and emotional support  - Support for care, protection, rest   - Understanding the community service delivery system   - Introduction of cooperation with community services | - Community-based   - A panel of people with disabilities representing disability-related institutions and organizations in the community introduces the following examples:   - How to find community-connected resources and working with the community - How community services can comprehensively intervene for people with disabilities through multidisciplinary cooperation, such as healthcare workers, therapists, and social workers | [26, 28] |
| 12 | Meeting people with disabilities in the community | - Students should have the attitude and values of living with people with disabilities, obtained through understanding disabilities through direct encounters with people with disabilities in the community. | Attitude | - Students are confident in dealing with people with disabilities. | - Meeting people with disabilities in the community   - Volunteer activities at disability-related organizations, such as the Welfare Center for People with Disabilities | - Community-based   - Direct encounter and activities with people with disabilities in the community | [44] |
|  |  |  | Skills | - Students exhibit improved abilities to interact with people with disabilities applying appropriate etiquette for disability type. |  |  |  |
| 13 | Research related to people with disabilities | - Students should understand the barriers to medical care access that people with disabilities in South Korea face and should think comprehensively about solutions. | Knowledge | - Students can explain the measures to improve policy for facilitating access to medical care for people with disabilities. | - Before conducting research, students are introduced to various sources and open data lists for disability research. - Students can develop relevant research topics and design research methods. | - School-based   - Submitting a report on the research topic and research methods   - Community-based   - If necessary, researching cooperation with disability-related organizations and people with disabilities | [45] |
|  |  |  | Attitude | - Students have an empathetic attitude towards people with disabilities experiencing an inconvenience while using medical services. |  |  |  |

**-** Care and Communication for Patients with Disabilities Course (Final Draft)

|  | Topic | Necessity | Area | Goal | Content | Method | Reference |
| --- | --- | --- | --- | --- | --- | --- | --- |
| 1 | What is communication? | - Students should understand the general principles and basic structure of communication and should be able to apply basic communication skills. | Knowledge | - Students can explain the basic concepts and principles of proper communication. - Students know verbal and non-verbal communication and can explain the conditions for success in verbal communication. - Students can explain how to communicate effectively. | - Communication mechanism - Conditions for communication to take place - Improper communication - Precautions for successful communication - Useful communication skills   - Listening with attention - Inducing reaction - Clarification   - Non-verbal skills   - Space adjustment: ensuring a proper distance and meeting patient’s eye level | - School-based   - Lectures on the principles and basic structure of communication as well as basic communication skills  - Discussion of factors of successful and unsuccessful communications after watching a video showing general conversation scenes  - Experiencing the roles of doctors and patients through role-plays | [26, 31] |
|  |  |  | Skills | - Students can apply the basic skills for effective communication. |  |  |  |
| 2 | Building rapport with patients with disabilities | - Doctors with good rapport-building skills can establish satisfactory relationships with patients and cope better with emotionally challenging situations. | Knowledge | - Students can explain how to establish basic relationships with patients with disabilities. | - Rapport formation - Empathy skills - Expressing personal support - Respect skills | - School-based   - Lectures on how to establish basic relationships with patients with disabilities, and watching a video of model practices  - Experiencing the roles of doctors and patients through role-plays |  |
|  |  |  | Skills | - Students can use the skills to establish basic relationships during conversations with patients with disabilities. |  |  | [24, 26] |
| 3 | Communication with patients with disabilities: type Ⅰ  (patients with visual impairment) | - An understanding of communication methods during treatment is required for smooth communication with patients with visual impairment. | Attitude | - Students are confident in treating people with visual impairment. | - Communication with patients with visual impairment - The following contents should be included:   - How to communicate during treatment (stating one's name and role, clearly and concisely explaining things or situations, explaining in detail what kind of examination will be performed at each stage of treatment and examination, explaining the examination site and the contact part in advance)  - Preparation of documents and information  - Preparation of assistive technology devices   - A role-play using the following example:   - During an abdominal examination of a patient with visual impairment, the doctor asks the patient to take a position suitable for the abdominal examination and proceeds with the examination. | - School-based   - Lectures on communication with patients with visual impairment  - Presenting specific cases and experiencing the roles of doctors and patients through role-plays  - Discussion and sharing of feelings from role-play experiences | - |
|  |  |  | Skills | - Students exhibit improved communication skills with patients with visual impairment. |  |  |  |
| 4 | Communication with patients with disabilities: type Ⅱ  (patients with hearing impairment) | - An understanding of communication methods during treatment is required for smooth communication with patients with hearing impairment. | Attitude | - Students are confident in treating people with hearing impairment. | - Communication with patients with hearing impairment - The following contents should be included:   - It is necessary to check whether the patients use a hearing aid or sign language.  - Avoiding yelling assuming that the patients are unable to hear  - Talking to one person at a time  - Using facial expressions and gestures  - If the patients do not understand the content of the conversation, delivering the same content in a different manner (audible material, written material)  - Using hearing assistive technology devices  - Supporting communication with patients with hearing impairment to the extent desired by the patients in the presence of a sign language interpreter (patient rights must be granted)   - A role-play using the following example:   - Measuring the blood pressure of a patient with hearing impairment. | - School-based   - Lectures on communication with patients with hearing impairment  - Presenting specific cases and experiencing the roles of doctors and patients through role-plays  - Discussion and sharing of feelings from role-play experiences | [29, 30, 31, 32] |
|  |  |  | Skills | - Students exhibit improved communication skills with patients with hearing impairment. |  |  |  |
| 5 | Communication with patients with disabilities: type Ⅲ  (patients with developmental disorders) | - An understanding of communication methods during treatment is required for smooth communication with patients with developmental disorders. | Attitude | - Students are confident in treating people with developmental disorders. | - Communication with patients with developmental disorders - The following contents should be included:   - Communication methods during treatment (questioning neutrally, asking the same content in different forms, using yes/no questions, talking directly with the patient, seeking patient consent from the patient’s companion while directing the gaze or gestures towards the patient)  - Abnormal behavior (challenging behavior) of patients with developmental disabilities and its meaning  - Responding to challenging behavior that does not infringe on the human rights of patients with developmental disorders   - Students discuss the appropriate behavior of the doctor in the following cases:   - In a situation where local anesthesia must be performed on a patient with a developmental disorder, the patient suddenly becomes angry and exhibits self-harming behavior. What should the doctor do at this time? | - School-based   - Lectures on communication with patients with developmental disorders  - Discussion of appropriate behavior of a doctor after presenting a specific example   - Community-based   - Special lectures from healthcare workers who often deal with people with developmental disorders and patients with developmental disorders | [11, 33, 36] |
|  |  |  | Skills | - Students exhibit improved communication skills with patients with developmental disorders. |  |  |  |
| 6 | Patient consent | - For patient safety and legal and ethical considerations, patients must be enabled to make autonomous decisions and consent based on sufficient information provided for easy understanding before an examination or procedure, and the entire process must be recorded. | Knowledge | - Students can explain the ethical importance of informed consent. - Students can list the information that must be provided to obtain informed consent. | - What is informed consent? - The ethical and legal importance of informed consent - Obtaining consent and communication - Components of informed consent - Considering decision-making skills   - Presenting an example of obtaining consent for using computer tomography contrast media from a patient with a mild intellectual disability and an IQ of 65 for a discussion of the procedure to obtain informed consent   - Information to be provided to the patient - Practical communication skills - Obtaining consent for exceptional circumstances | - School-based   - Lectures on the ethical importance of informed consent and information to be provided when obtaining informed consent  - Discussion of how to proceed with the informed consent process for patients with insufficient decision-making ability, such as patients with intellectual disabilities | - |
| 7 | Basic principles of treatment of patients with disabilities | - Understanding \ the basic principles of treating patients with disabilities in the medical domain is required for smooth interaction with patients with disabilities. | Knowledge | - Students can explain the attitude of healthcare workers in treating patients with disabilities. | - Basic principles for the treatment of patients with disabilities in the medical domain   - Avoiding restricting, excluding, separating, or rejecting patients with disabilities in medical practice  - Considering sex, type, severity, and characteristics of disability for medical practice and research of patients with disabilities  - Providing medical support services suitable for the type, severity, and characteristics of disabilities | - School-based   - Lecture on the basic principles of treatment for patients with disabilities  - Discussion after watching a related video (effective and ineffective treatment examples) | [11, 25, 34] |
| 8 | Treatment of patients with disabilities: type Ⅰ | - Understanding treatment methods for patients with disabilities is required for smooth interaction with patients with disabilities. | Knowledge | - Students can explain the basic principles of treatment for patients with physical and mental disabilities. | - Treatment methods according to the general characteristics of patients with physical and mental disabilities   - General physical examination  - Image inspection, such as X-ray  - Endoscopy and invasive procedures  - Types of examination equipment for disability characteristics   - How to move wheelchair ↔ examination table for patients using a wheelchair | - Community-based   - Lectures by experts who treat patients with disabilities  - Practice on how to move patients using a wheelchair | [41] |
|  |  |  | Skills | - Students can move patients using a wheelchair. |  |  |  |
| 9 | Treatment of patients with disabilities: type Ⅱ | - Understanding treatment methods for patients with disabilities is required for smooth interaction with patients with disabilities. | Knowledge | - Students can explain the basic principles of treatment for patients with visual and hearing impairments. - Students can explain the basic principles of treatment for patients with intellectual disabilities and mental disorders. | - Treatment methods according to the general characteristics of patients with visual and hearing impairments and patients with intellectual disabilities and mental disorders   - General physical examination  - Image inspection, such as X-ray  - Endoscopy and invasive procedures | - Community-based   - Lectures by experts who treat patients with disabilities | [27] |
| 10 | Meeting patients with disabilities | - Students should have the attitude and values of living with people with disabilities, obtained through understanding disabilities through direct encounters with people with disabilities in the community. | Attitude | - Students are confident in treating people with disabilities. | - Meeting people with disabilities in the community   - Providing medical service activities in connection with disability-related institutions and organizations in the community  - Interaction with people with disabilities using appropriate etiquette based on disability type  - Measuring blood pressure and giving health advice applying the basic treatment principles based on disability type  (※Medical service activities should be conducted with an advisor or community doctor who can manage and supervise students.) | - Community-based   - Direct encounter and activities with people with disabilities in the community  - Providing medical service activities in connection with disability-related institutions and organizations in the community | [26, 33, 44] |
|  |  |  | Skills | - Students exhibit an improved ability to interact with people with disabilities by applying the basic principles of treatment based on disability type. |  |  |  |
| 11 | Mock interviews using standardized patients Ⅰ  (patients with visual impairment) | - Students should indirectly experience the treatment of people with disabilities in a safe and educational environment that is close to reality through mock interviews using standardized patients. | Attitude | - Students have an altruistic attitude towards providing patient-centered care for patients with disabilities. - Students are confident in dealing with people with disabilities. | - Using a scenario of a patient with visual impairment who visits an outpatient clinic due to increased urine volume, students interview the patient and perform a basic physical examination. | - School-based   - Clinical performance skill tests, such as the Objective Structured Clinical Examination (OSCE) and the Clinical Performance Examination (CPX)  - Evaluation through scoring - Observation and feedback through video recording  - Self-report and discussion by students | [9] |
|  |  |  | Skills | - Students exhibit improved face-to-face communication skills with patients with disabilities. - Students exhibit improved general basic physical examination skills for patients with disabilities. |  |  |  |
| 12 | Mock interviews using standardized patients Ⅱ  (patients with intellectual disabilities) | - Students should indirectly experience the treatment of people with disabilities in a safe and educational environment that is close to reality through mock interviews using standardized patients. | Attitude | - Students have an altruistic attitude towards providing patient-centered care for patients with disabilities. - Students are confident in dealing with people with disabilities. | - Using a scenario of a patient with mild intellectual disability who visits a clinic due to cold symptoms, students interview the patient and perform a basic physical examination. | - School-based   - Clinical performance skill tests  (OSCE/CPX)  - Evaluation through scoring - Observation and feedback through video recording  - Self-report and discussion by students | [9] |
|  |  |  | Skills | - Students exhibit improved face-to-face communication skills with patients with disabilities. - Students exhibit improved general basic physical examination skills for patients with disabilities. |  |  |  |

**Supplementary File 2.** Education contents that were not selected by experts

- Basic Introductory Course

| Topic | Necessity | Area | Goal | Content | Method |
| --- | --- | --- | --- | --- | --- |
| Disability Experience Education II | - Students can have an empathetic attitude towards people with disabilities through sign language education and auditory hallucination simulation | Knowledge | - Students are able to use basic sign languages. | - Basic sign language education   - Numbers and basic medical terms (sick, dizzy, etc.)   - Auditory hallucination simulation | - Community-based   - Special lectures by Korean sign language interpreters with disabilities   - School-based   - Auditory hallucination simulation  - Presentation of feelings about sign language education and simulation hallucination experience activities |

**-** Care and Communication for Patients with Disabilities Course

| Topic | Necessity | Area | Goal | Content | Method |
| --- | --- | --- | --- | --- | --- |
| Patient Consent II  (Consent for CT scan) | - It is important in terms of patient safety and legal and ethical aspects to enable the patients to make autonomous decisions and consents based on enough information provided for easy understanding prior to an examination or procedure, and to record the entire process. | Skills | - Students are able to provide easy-to-understand information about an examination or procedure and can obtain voluntary consent from patients. - Students are able to properly use the consent form for obtaining informed consent. | - Students follow a series of processes for obtaining consent forms according to the suggested scenarios.   - Scenario example: Obtaining consent for using CT contrast media from a patient with a mild intellectual disability with an IQ of 65   - Students proceed with the process to obtain an informed consent, including the following:   - Explanation of the test details (name, risk, etc.)  - Checking the understanding of the patient and provide opportunities to ask questions  - Voluntary patient consent and written confirmation  - Explain effectively according to the level of the patient (considering the level of decision-making ability and intellectual level, using images, etc.) | School-based  - Dividing into teams to conduct role-plays while providing feedback through peer evaluation |
| Patient Consent III  (Organ Donation) | - It is important in terms of patient safety and legal and ethical aspects to enable the patients to make autonomous decisions and consents based on enough information provided for easy understanding, and to record the entire process. | Skills | - Students are able to provide easy-to-understand information about organ donation and can obtain voluntary consent from patients. - Students make sure that organ donation is not due to family pressure or external pressure while respecting the patient's wishes as much as possible. | - Students follow a series of processes for obtaining consent forms according to the suggested scenarios.   - Scenario example: Obtaining consent for kidney from a patient with a mild intellectual disability with an IQ of 65   - Students proceed with the process to obtain an informed consent, including the following:   - Explanation of the test/surgery details (name, risk, etc.)  - Checking the understanding of the patient and provide opportunities to ask questions  - Voluntary patient consent and written confirmation  - Explain effectively according to the level of the patient (considering the level of decision-making ability and intellectual level, using images, etc.) | School-based  - Dividing into teams to conduct role-plays while providing feedback through peer evaluation |
